# 

**Published:** 1963-10

**Authors:** 


BRISTOL MEDICO-CHIRURGICAL JOURNAL

*(Incorporating the Medical Journal of the South-West)*

Established 1883

VOL. 78 (iv) October 1963 NO. 290

### Contents

| | | |
|---|---|---|
| MEDICINE IN NORTH AMERICA | *J. Apley* | 109 |
| HAEMATURIA AND OFFICE UROLOGY | *H. Guerrier* | 114 |
| A FOETUS WITH DOWN'S SYNDROME | *M. B. Wingate* | 121 |
| LAST CONSCIOUS THOUGHTS | *G. De M. Rudolf* | 125 |
| CLASSIFICATION OF JUVENILE DELINQUENTS | *F. Bodman* | 127 |
| OBITUARY | | 131 |
| SOUTH WEST SURGEONS | | 132 |
| WESSEX RAHERE CLUB | | 132 |
| EXAMINATION RESULTS | | 133 |
| CORRESPONDENCE | | 134 |

*PUBLISHED QUARTERLY*

*ANNUAL SUBSCRIPTION* 16/- *POST FREE*

PUBLISHED UNDER THE AUSPICES OF THE
BRISTOL MEDICO-CHIRURGICAL SOCIETY

### *Editorial Committee*

Dr. N. J. Brown, Southmead Hospital, Bristol. *Joint Editor.*

Dr. A. B. Raper, Department of Pathology, Bristol Royal Infirmary. *Joint Editor.*

Dr. J. Macrae, Ham Green Hospital.

Dr. R. C. Wofinden, Central Clinic, Bristol.

Mr. A. E. S. Roberts, Medical Library, University of Bristol. *Secretary.*

*Local Correspondents*

*Bath* . . Mr. A. D. Bateman, 3 The Circus, Bath.

*Exeter* . . Dr. L. N. Jackson, Mount Jocelyn, Crediton, Devon.

*Gloucester* . Dr. R. F. Jarrett, 9 College Green, Gloucester.

*Plymouth* . Dr. C. R. Croft, Lexden, Hartley Av., Plymouth.

*Taunton* . Dr. M. McAlley, 1 The Crescent, Taunton.

*Torquay* . Dr. A. Robinson Thomas, 20 Devon Square, Newton Abbot, Devon.

*Truro* . . Dr. F. D. M. Hocking, St. Andrews, Perranporth, Cornwall.

*Yeovil* . . Mr. J. A. Vere Nicoll, Knapp House, Yeovil, Somerset.

### NOTICE TO CONTRIBUTORS

Original articles are invited, provided they have not been published elsewhere. Notes of forthcoming events, meetings held, degrees conferred, staff appointments, and other local news are welcomed. The editorial committee reserves the right to make literary corrections.

Illustrations should always be supplied ready for reproduction. All re-touchings or other operations required will be charged to the author. Line drawings should be drawn in Indian ink. We regret that we must ask authors to pay for making blocks, if this is necessary.

J. W. ARROWSMITH LTD., WINTERSTOKE ROAD,
BRISTOL.

### *Examination Results*

*M.R.C.P.* (*London*): D. B. Evans, N. H. Stentiford.
*Diploma of Industrial Health* (*England*): J. L. Elliott.

### UNIVERSITY OF BRISTOL

*M.D.:* G. A. J. Ayliffe, M. O. Symes.

### *M.B., CH.B.*

*Second Class Honours*

Duffin, Gwendolen Mary
Paget, Richard James

*Pass*

Ainsworth, Patricia

Bott, Susan Elizabeth

Bridges, Diana Patricia

Chu, Kim Leung Robert

Cole, Anthony Paul

Davies, Aeron Gwyn

Davies, Peter Llewelyn

Davis, Felicity Ann

Dickens, Anthony John Gilmore

Dovey, Michael Sydney

Fuller, Alison Jean

Greene, Malcolm John

Hilton, Jacqueline

Huggins, Mary Winifred

Hunt, Phyllis Gillian

James, Verity Edwina

Johnson, Paul

Khoo, Eu-Eng

Lawrie, Brian William

Leong, Siew Mun

Lewis, David

Lloyd, Anthony Mills

Louisy, Coventry Laurent

Louisy, Sheila Irene

Marval, Michael John

Mazhar, Mahmood Mohamed

Pitt-Aikens, Tom

Plant, Patricia Anne

Polkey, Charles Edward

Pugsley, David John

Sempa, Alexandra Vera

Seymour, Richard Nicholas

Stephens, Michael Redford

Stevenson, Paul Anthony

Vahala, Karel Vaclav Jiri

Warr, Edward Ernest

White, John Michael Roger

Whitehead, Philip John

Williamson, Ronald

